# The association between malnutrition risk and revised Edmonton Symptom Assessment System (ESAS-r) scores in an adult outpatient oncology population: a cross-sectional study

**DOI:** 10.1186/s41687-024-00750-8

**Published:** 2024-07-12

**Authors:** Katherine McLay, Nicole Stonewall, Laura Forbes, Christine Peters

**Affiliations:** 1 Temerty Faculty of Medicine, University of Toronto, 1 King’s College Circle Medical Sciences Building, Toronto, ON M5S 1A8 Canada; 2 Waterloo Wellington Regional Cancer Program, Grand River Regional Cancer Centre, Kitchener, ON Canada; 3 Department of Family Relations and Applied Nutrition, University of Guelph, Guelph, ON N1G 2W1 Canada

**Keywords:** Nutrition, Oncology, Cancer care, ESAS, Patient-reported outcomes, Quality of life

## Abstract

**Background:**

Cancer-associated malnutrition is associated with worse symptom severity, functional status, quality of life, and overall survival. Malnutrition in cancer patients is often under-recognized and undertreated, emphasizing the need for standardized pathways for nutritional management in this population. The objectives of this study were to (1) investigate the relationship between malnutrition risk and self-reported symptom severity scores in an adult oncology outpatient population and (2) to identify whether a secondary screening tool for malnutrition risk (abPG-SGA) should be recommended for patients with a specific ESAS-r cut-off score or group of ESAS-r cut-off scores.

**Methods:**

A single-institution retrospective cross-sectional study was conducted. Malnutrition risk was measured using the Abridged Patient-Generated Subjective Global Assessment (abPG-SGA). Cancer symptom severity was measured using the Revised Edmonton Symptom Assessment System (ESAS-r). In accordance with standard institutional practice, patients completed both tools at first consult at the cancer centre. Adult patients who completed the ESAS-r and abPG-SGA on the same day between February 2017 and January 2020 were included. Spearman’s correlation, Mann Whitney U tests, receiver operating characteristic curves, and binary logistic regression models were used for statistical analyses.

**Results:**

2071 oncology outpatients met inclusion criteria (mean age 65.7), of which 33.6% were identified to be at risk for malnutrition. For all ESAS-r parameters (pain, tiredness, drowsiness, nausea, lack of appetite, shortness of breath, depression, anxiety, and wellbeing), patients at risk for malnutrition had significantly higher scores (*P* < 0.001). All ESAS-r parameters were positively correlated with abPG-SGA score (*P* < 0.01). The ESAS-r parameters that best predicted malnutrition risk status were total ESAS-r score, lack of appetite, tiredness, and wellbeing (area under the curve = 0.824, 0.812, 0.764, 0.761 respectively). Lack of appetite score ≥ 1 demonstrated a sensitivity of 77.4% and specificity of 77.0%. Combining lack of appetite score ≥ 1 with total ESAS score > 14 yielded a sensitivity of 87.9% and specificity of 62.8%.

**Conclusion:**

Malnutrition risk as measured by the abPG-SGA and symptom severity scores as measured by the ESAS-r are positively and significantly correlated. Given the widespread use of the ESAS-r in cancer care, utilizing specific ESAS-r cut-offs to trigger malnutrition screening could be a viable way to identify cancer patients at risk for malnutrition.

## Background

Malnutrition has been defined as a state of altered nutritional status that is associated with increased risk of adverse clinical events [1]. Cancer-associated malnutrition is a complex and multifactorial process distinct from malnutrition due to simple starvation. It is characterized by a combination of reduced food intake and metabolic derangements caused by the cancer itself and/or anticancer therapies [2–4]. The variable presence of interacting factors such as inflammatory and catabolic mediators, symptoms impairing food intake, mass effects such as tumour obstruction of the gastrointestinal tract, and impaired organ function contribute to cancer patients being among the most malnourished of all patient groups [2, 5]. The estimated prevalence of malnutrition in cancer patients ranges from 30 to 87% across studies, with gastroesophageal and pancreatic cancers carrying the greatest risk of malnutrition [1, 6–8].

It is well-established that nutritional status and weight loss are important prognostic indicators in cancer patients. Malnutrition in the context of cancer is associated with symptom severity [9, 10], functional status [10–13], systemic inflammation [14, 15], response to cancer treatments [2, 16–18], quality of life [10, 16, 19], length of hospital stay [13, 20–23], cost of care [13, 20, 22] and survival [13, 15, 19, 23–25]. Weight loss in cancer patients is independently associated with reduced treatment tolerance, quality of life, functional capacity, and survival [8, 18, 26–29]. Cancer patients who maintain their weight have been shown to demonstrate a survival benefit ranging from 14 to 51% over patients who lose weight, with loss of as little as < 5% of body weight being associated with worse prognosis [27]. Malnourished cancer patients may develop cachexia, a complex metabolic syndrome characterized by loss of muscle mass and variable loss of fat due to pathologically increased protein catabolism [6]. Cachexia is associated with significant morbidity and mortality; it is estimated that 20% of cancer deaths are attributable to cachexia [6, 30]. Cancer patients with cachexia are invariably malnourished, and this condition cannot be fully reversed by nutritional support [6, 30].

Nutritional interventions tailored to patients’ needs have been shown to reduce negative consequences from cancer-associated malnutrition and improve quality of life, functional status, weight gain, treatment tolerance, and overall survival [24, 31–33]. Due to the characteristic progression of cancer-associated malnutrition to irreversible cachexia, nutritional therapy derives the highest patient benefit if implemented early in the cancer treatment course [16, 24, 32–37]. As such, it is important to identify individuals who are malnourished or at risk of malnutrition so that indicated nutritional therapies can be initiated in a timely manner.

Malnutrition screening aims to identify cancer patients who are malnourished or at risk of malnutrition. International evidence-based guidelines broadly agree that nutritional screening for cancer patients should be conducted at diagnosis and routinely repeated through each stage of treatment [3, 38–41]. However, there is a lack of research and consensus surrounding the optimal timing, triggers, and tools for ongoing nutritional screening and assessment [3, 36, 37, 42, 43]. This has contributed to cancer-associated malnutrition being underrecognized and undertreated [16, 37, 43, 44]. An international prospective audit conducted across 11 centres in Canada, Australia, Italy, the Netherlands, and the United States found that while all centres incorporated nutritional screening and assessment at some point during care of cancer patients at high nutritional risk, adherence to evidence-based guidelines regarding nutritional monitoring and evaluation of patients throughout treatment ranged from 13 to 66%. The same study found that validated tools and instruments for nutritional screening were used across only 64% of the centres [38]. These findings highlight that although international guidelines agree on its necessity, delivery of malnutrition screening in clinical practice poses challenges and further research on nutritional cancer care pathways is needed.

A potential approach to this problem involves utilizing the revised Edmonton Symptom Assessment System (ESAS-r) to trigger malnutrition screening. The ESAS-r is a patient-completed tool used for symptom screening and longitudinal monitoring in oncological care [45]. Patients are prompted to score nine oncological symptoms on a numeric rating scale and indicate where on their body they are experiencing pain. The symptoms included are pain, tiredness, drowsiness, nausea, lack of appetite, shortness of breath, depression, anxiety, and wellbeing. The ESAS-r is deliberately concise to minimize patient burden [45]. This tool is intended to be used for symptom screening within a larger clinical assessment, where concerning symptoms are identified and followed up with a more in-depth assessment [45]. Therefore, as it is employed routinely for symptom monitoring, the ESAS-r is well positioned as a potential means to identify cancer patients requiring nutritional evaluation.

The best available method for assessing nutritional status in oncology patients is considered to be the Patient Guided Subjective Global Assessment (PG-SGA) [37, 46–48]. The PG-SGA was adapted from the nutritional assessment instrument SGA for specific use in oncology. It is composed of four sections assessing aspects of nutritional status including weight, intake, symptoms, functional status, disease state, metabolic stress, and physical examination [48]. Administration of the PG-SGA requires time and trained staff, which is a barrier to implementation and adherence in busy outpatient settings [37, 46, 49]. The abridged PG-SGA (abPG-SGA), which is shorter and forgoes the physical examination, has therefore been employed as a more feasible option for nutritional screening in outpatient oncology populations [46, 49]. The abPG-SGA, known also as the PG-SGA SF, is completed entirely by the patient and has been shown to perform comparably to the PG-SGA in an outpatient setting [46, 47, 49].

The aims of our study are to (1) investigate the relationship between malnutrition risk and ESAS-r scores at first consult in an adult oncology population using patient-reported outcome measures and (2) identify whether a secondary screening tool for malnutrition risk (abPG-SGA) should be prompted for a specific ESAS-r cut-off score or group of ESAS-r cut-off scores completed by outpatient oncology patients.

## Methods

**Fig. 1:**
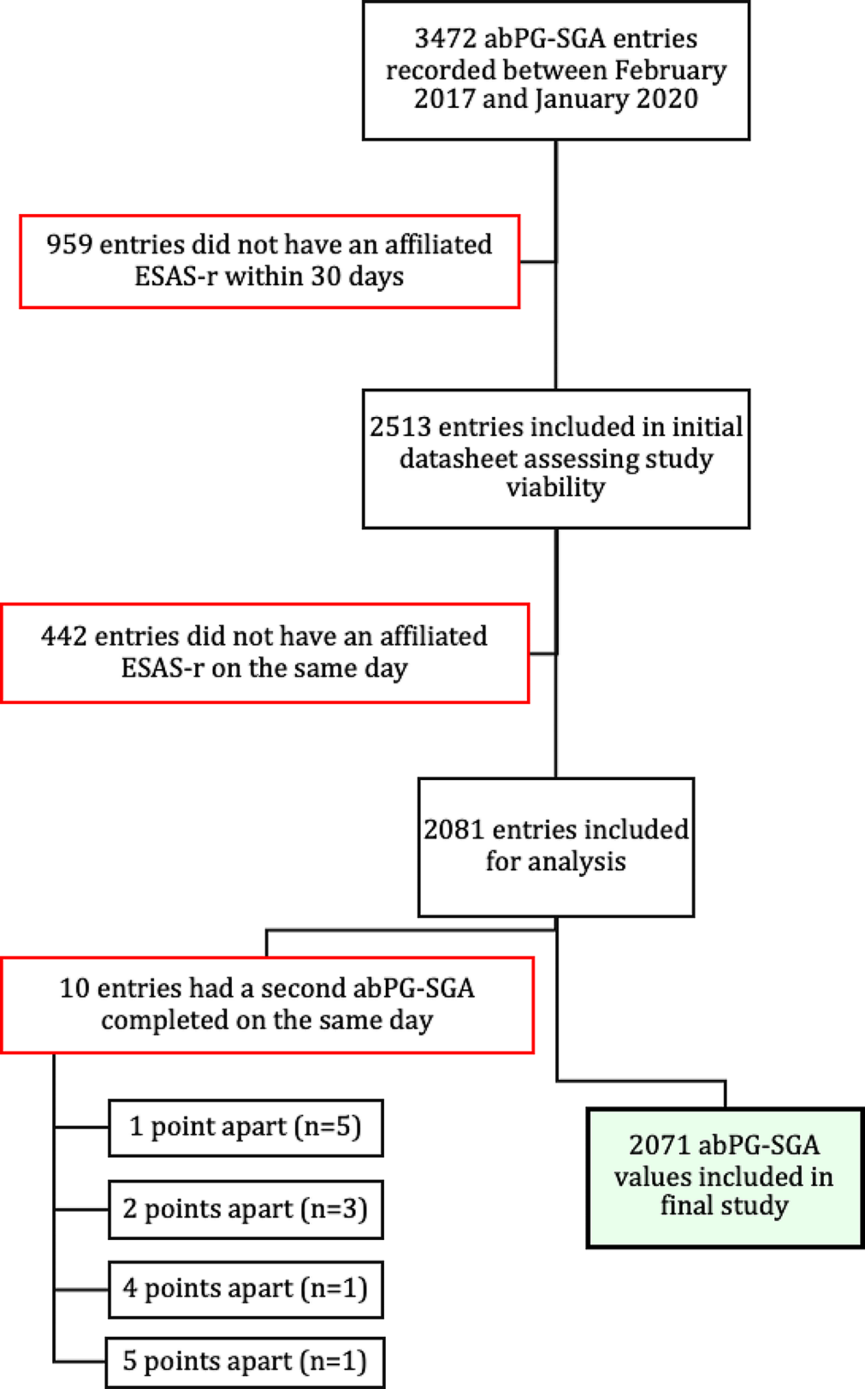
Flowchart depicting patients included in study analysis

This is a retrospective, cross-sectional study conducted using existing patient databases from Grand River Hospital and Cancer Care Ontario. A total of 3472 consecutive oncology outpatients aged 18 and older referred to the Grand River Regional Cancer Centre (GRRCC) completed the malnutrition screening tool (abPG-SGA) at first consult between February 2017 and January 2020. All patients who had a radiation or medical oncology consult were eligible for screening. Implementation of the abPG-SGA began in February 2017 in response to an internal quality review that found only 8% of cancer patients identified at nutrition risk at GRRCC were being referred to a dietitian for nutritional intervention. A database containing abPG-SGA scores for patients at first consult was subsequently created for quality review purposes. The ESAS-r tool is routinely completed by all cancer patients prior to their clinic visits at the GRRCC. For the purposes of assessing the viability of this study, a spreadsheet was created linking abPG-SGA scores with ESAS-r scores completed by the same patients at first consult. To obtain the most accurate associations between malnutrition risk and cancer symptoms, this cross-sectional study included patients who completed an ESAS-r on the same day as the abPG-SGA (*n* = 2071). For patients who completed two abPG-SGA scores on the date of first consult (*n* = 10), the higher of the two was included. The rationale for this is that in the context of malnutrition screening, over-screening patients who may not be malnourished or at risk for malnutrition is preferred to missing patients who are at risk and would benefit from nutritional intervention. Figure 1 shows the process for participant inclusion in this study. The study was approved by the Waterloo Wellington Research Ethics Board (formerly known as the Tri-Hospital Research Ethics Board, study identification number THREB # 2021 − 0733) and the University of Guelph Research Ethics Board (approval number #21-09-008).

### Assessing nutritional status

As part of the initial clinical consultation process at the GRRCC, patients were asked to complete the abPG-SGA tool for nutritional assessment. The abPG-SGA, although more labour intensive compared to other nutritional screening tools, was chosen for the GRRCC because of the scoring system that stratifies malnutrition risk level which is more clinically useful than a binary scoring system. The abPG-SGA has been shown to be practical and valid when employed in an outpatient oncology setting and performs comparably to the PG-SGA [46, 47, 49]. A study by Gabrielson et al. (*n* = 90) found that the abPG-SGA demonstrated 94% sensitivity and 78% specificity in predicting malnutrition status as assessed by the SGA, which was slightly lower than the PG-SGA (97% sensitivity, 72% specificity) and higher than the Malnutrition Screening Tool (81% sensitivity, 72% specificity) [46].

The abPG-SGA is scored from 0 to 36, and the patient’s score corresponds to recommendations for interdisciplinary intervention based on nutritional risk. Higher scores correspond to a greater risk of malnutrition, where scores of 4–8 indicate that multidisciplinary nutritional intervention is required, and scores of ≥ 9 indicate a critical need for improved symptom management and/or nutritional intervention [48]. For this study, patients were identified to be at risk of malnutrition if they received an abPG-SGA score of 6 or greater.

### Measurement of cancer symptom severity

Scores for nine oncological symptoms (pain, tiredness, drowsiness, nausea, lack of appetite, shortness of breath, depression, anxiety, and well-being) were measured using the ESAS-r. The ESAS-r is a patient-reported outcome measure that captures how the patient is feeling at the time of completing the tool and is routinely administered throughout cancer care as established by standard procedures at GRRCC. Patients are asked to complete an ESAS-r at each clinic visit. A database containing ESAS-r scores and demographic information for patients at the GRRCC was obtained from Ontario Health (Cancer Care Ontario).

The ESAS-r was developed through a collaboration between Alberta Health Services, Covenant Health in Alberta, and the University of Alberta as an evidence-based revision to the Edmonton Symptom Assessment System (ESAS). The ESAS-r retains the core elements of the ESAS while introducing minor changes intended to improve clarity [45]. The ESAS has been widely psychometrically validated and translated into over 30 languages, and the ESAS-r has been shown to be reliable and valid in an outpatient oncology population [45, 50, 51]. Compared to the ESAS, the ESAS-r has been shown to be significantly easier to understand and preferred by patients [45, 52].

### Demographic variables

Data for this study also included demographic information from the patient databases used. These data included age, gender, cancer type and cancer staging. However, cancer staging was not available for a significant number of patients at first consult, so this information was not included in the present analysis.

### Statistical analyses

Statistical analyses were conducted using IBM SPSS Statistics software (version 28.0: SPSS, Chicago, I.L., USA). Correlation analyses to examine the relationship between nutrition risk (abPG-SGA) and ESAS-r symptom scores were conducted using Spearman’s rho. Using the established threshold score of ≥ 6 on the abPG-SGA, patients were allocated into one of two groups: at risk for malnutrition or not at risk for malnutrition. As neither the abPG-SGA nor ESAS-r scores were normally distributed, nonparametric Mann-Whitney U tests were used for analysis between malnutrition risk groups. To examine the ability of ESAS-r symptom scores to predict risk status for malnutrition, receiver operating characteristic (ROC) curves were generated and the sensitivity and specificity of different ESAS-r symptom score cut-offs for identifying malnutrition risk were calculated. In the context of nutritional screening, high sensitivity (correctly identifying those at risk for malnutrition) takes precedence over high specificity (correctly identifying those not at risk for malnutrition) [49]. As such, given that sensitivity values for each individual ESAS-r symptom fell below 56% at the cut off of > 4, sensitivity and specificity values are reported for thresholds ranging from > 0 to > 3. The area under the curve (AUC) was used to summarize the overall accuracy of the ESAS-r symptom scores in predicting malnutrition risk status (identified at risk for malnutrition or not identified at risk for malnutrition). To determine what combination of ESAS score parameters predicted malnutrition risk, binary logistic regression models were created. ESAS parameters were entered into models using forward step-wise selection. Demographic characteristics were not controlled for in the models as only age and gender were available and neither were related to malnutrition risk in this population. One model was created that included all participants. Models stratified by gender and stratified by cancer type (GI cancers vs. non-GI cancers) were also created. The rationale for stratifying by cancer type with GI cancers vs. non-GI cancers is that GI cancers such as pancreatic and gastroesophageal have been shown to carry a higher risk of malnutrition in the literature [7]. Further, as GI cancers can have a more direct impact on the alimentary canal, we aimed to evaluate whether the ESAS symptoms most predictive of malnutrition risk were different in this population compared with non-GI cancers. The final models presented were those that best predicted malnutrition without evidence of suppressor effects. Statistical significance for the analyses was reported at the *P* < 0.05 level (two-tailed).

## Results

The study sample comprised 2071 patients (785 men, 1286 women) with a mean age of 65.7 ± 13.7. The most prevalent types of cancers were breast (29.0%), gastrointestinal (20.3%), haematological (19.3%), and lung (12.3%) cancers. In terms of cancer stage, the highest proportion of patients fell into the ‘not staged’ category (20.4%); this is expected as patient information was generally captured early in the treatment trajectory, primarily at first consult with a medical or radiation oncologist. The remaining cancer staging categories (stages 1–4, not stageable) were approximately evenly represented, with a smaller proportion of patients falling into the categories of stage 0 (2.4%), stage unknown (0.7%), and stage not valid (0.1%). There were no missing values for any demographic parameters. Characteristics of the study sample are reported in Table 1.

**Table 1:** Characteristics of the study sample (*N* = 2071)

Characteristic	*n* (%)
Gender	
Male	785 (37.9%)
Female	1286 (62.1%)
Age (yrs)*	
18–39	97 (4.7%)
40–59	502 (24.2%)
60–79	1150 (55.5%)
80+	322 (15.5%)
Type of cancer	
Breast	600 (29.0%)
Gastrointestinal	421 (20.3%)
Haematological	399 (19.3%)
Lung	254 (12.3%)
Skin	142 (6.9%)
Genitourinary	109 (5.3%)
Gynaecological	77 (3.7%)
Sarcoma	23 (1.1%)
Primary unknown	19 (0.9%)
Central nervous system	12 (0.6%)
Head and neck	10 (0.5%)
Other cancers	3 (0.1%)
Endocrine	2 (0.1%)
Stage of cancer	
Stage 0	49 (2.4%)
Stage 1	343 (16.6%)
Stage 2	311 (15.0%)
Stage 3	280 (13.5%)
Stage 4	362 (17.5%)
Not stageable	288 (13.9%)
Not staged	422 (20.4%)
Not valid	2 (0.1%)
Valid, stage unknown	10 (0.5%)
Unknown	4 (0.2%)

*Age mean ± standard deviation = 65.68 ± 13.66 yrs

From the sample of 2071 patients, 696 (33.6%) were identified as at risk for malnutrition (abPG-SGA ≥ 6). Mann-Whitney U analyses indicated that all ESAS-r symptom scores were higher for patients identified at risk for malnutrition than for patients not identified at risk for malnutrition. For each ESAS-r parameter, the difference between nutritional risk groups was statistically significant with *P* < 0.001. These analyses are presented in Table 2.

**Table 2:** Results of Mann-Whitney U-test on the differences in ESAS-r symptom scores between patients identified at risk for malnutrition and patients not identified at risk of malnutrition (*N* = 2071)

	Patients not identified at risk for malnutrition (*n* = 696)	Patients identified at risk for malnutrition (*n* = 1375)	
	Median (IQR)	Median (IQR)	*p*
Pain	0 (1)	2 (5)	<0.001
Tiredness	1 (4)	5 (4)	<0.001
Nausea	0 (0)	0 (2)	<0.001
Depression	0 (1)	1 (4)	<0.001
Anxiety	1 (4)	3 (5)	<0.001
Drowsiness	0 (2)	2 (5)	<0.001
Lack of appetite	0 (0)	3 (5)	<0.001
Wellbeing	1 (3)	4 (4)	<0.001
Shortness of breath	0 (1)	1 (4)	<0.001

Correlation analyses using Spearman’s rho found that malnutrition risk (abPG-SGA score) was positively and significantly correlated with symptom severity for all nine symptoms captured by the ESAS-r. Lack of appetite, wellbeing, and tiredness were found to have the strongest correlations with malnutrition risk (ρ = 0.602, 0.514, 0.510, respectively). It is important to note that higher scores for the wellbeing parameter on the ESAS-r represent worse wellbeing (0 = best wellbeing, 10 = worst possible wellbeing), and higher scores for the lack of appetite parameter represent a more severe lack of appetite (0 = no lack of appetite, 10 = worst possible lack of appetite). The ESAS-r parameters that demonstrated the weakest correlations with malnutrition risk were anxiety (ρ = 0.293), shortness of breath (ρ = 0.311), and depression (0.322). As reported in Table 3, all correlations were statistically significant with *P* < 0.01 (two-tailed).

**Table 3:** Correlation between abPG-SGA score and ESAS-r scores using Spearman’s rho (*N* = 2071)

ESAS-*r* symptom scores	abPG-SGA score
Lack of appetite	0.602**
Wellbeing	0.514**
Tiredness	0.510**
Pain	0.426**
Drowsiness	0.399**
Nausea	0.361**
Depression	0.322**
Shortness of breath	0.311**
Anxiety	0.293**

Values are Spearman’s correlation coefficients (ρ)

**p < 0.01, 2-tailed

**Fig. 2:**
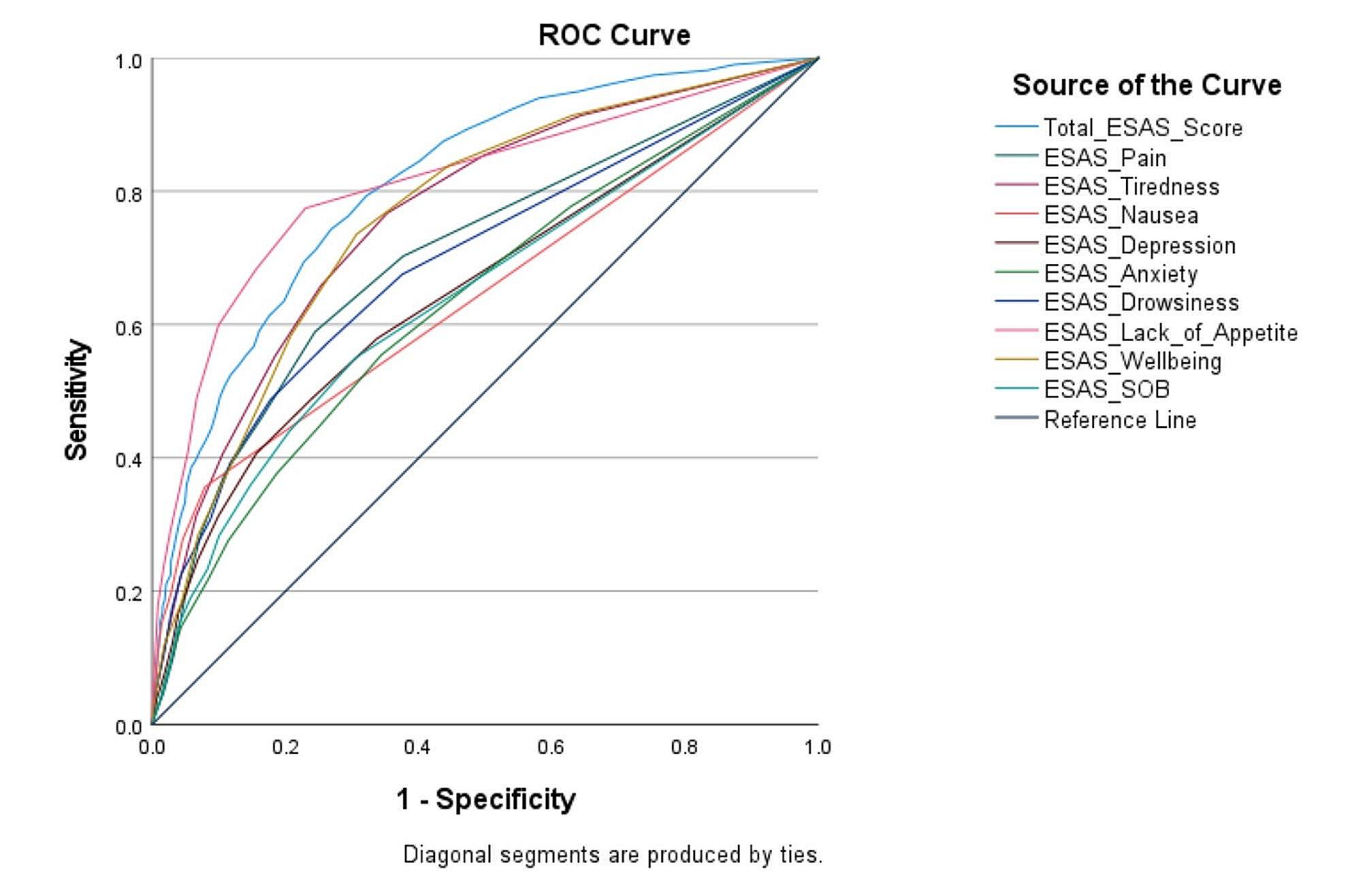
Receiver operating characteristic (ROC) curves for ESAS-r parameters in distinguishing between nutritional risk status

ROC analyses indicated that the ESAS-r parameters of total ESAS score, lack of appetite, tiredness, and wellbeing were most accurate at differentiating between patients identified at risk for malnutrition and patients not identified at risk for malnutrition (AUC = 0.824, 0.812, 0.764, and 0.761 respectively). Figure 2 depicts the ROC curves for each ESAS-r parameter. Sensitivity and specificity values in predicting nutritional risk status for each ESAS-r parameter are reported for the cutoff points of > 0, >1, > 2, and > 3 in Table 4.

**Table 4:** Receiver operating characteristic analysis for ESAS-r parameters in distinguishing between nutritional risk status at different cut-off values (*N* = 2071)

ESAS-r symptom	Area under the curve	Cut-off points	Sensitivity (%)	Specificity (%)
Lack of appetite	0.812			
		>0	77.4	77.0
		>1	68.2	84.4
		>2	59.9	90.0
		>3	49.0	93.3
Tiredness	0.764			
		>0	91.4	35.6
		>1	85.3	50.3
		>2	76.7	64.7
		>3	65.8	74.7
Wellbeing	0.761			
		>0	91.4	36.9
		>1	83.9	55.4
		>2	73.6	69.3
		>3	58.3	79.2
Pain	0.703			
		>0	70.3	62.3
		>1	58.9	75.6
		>2	46.3	83.4
		>3	37.6	89.1
Drowsiness	0.693			
		>0	67.5	62.5
		>1	57.3	73.6
		>2	48.7	82.3
		>3	39.2	88.3
Depression	0.651			
		>0	57.9	66.3
		>1	49.0	75.9
		>2	40.7	84.4
		>3	31.3	90.0
Nausea	0.643			
		>0	35.6	92.1
		>1	27.7	95.4
		>2	19.7	97.2
		>3	15.2	98.6
Shortness of breath	0.642			
		>0	55.0	69.5
		>1	44.0	79.4
		>2	35.9	85.2
		>3	28.3	90.0
Anxiety	0.635			
		>0	77.7	37.2
		>1	67.0	51.1
		>2	55.5	65.6
		>3	44.8	74.8
Total ESAS score	0.824			
		>0	99.0	12.7
		>1	98.1	16.7
		>2	97.4	24.7
		>3	96.1	31.1
		>4	95.0	36.0
		>5	94.0	42.0
		>6	92.4	45.8
		>7	90.8	49.2
		>8	89.4	52.6
		>9	87.5	56.3
		>10	84.5	59.9
		>11	82.8	63.7
		>12	80.9	65.4
		>13	79.3	67.9
		>14	76.3	70.5
		>15	74.3	73.2
		>16	71.3	75.4
		>17	69.4	77.2
		>18	65.9	79.1
		>19	63.5	80.2
		>20	61.4	82.4

Binary logistic regression analysis was conducted to determine which combination of ESAS-r score components would best predict malnutrition risk. As shown in Table 5, the combination of lack of appetite and total ESAS-r score correctly identified 90.9% of those with no nutrition risk and 55.6% of those with malnutrition risk.

**Table 5:** Binary logistic regression model to predict malnutrition risk based on ESAS-r symptom scores among adult patients with cancer

ESAS score symptoms	B	S.E.	Sig.	Odds ratio	95% CI
Lack of appetite	0.361	0.031	<0.001	1.435	1.351–1.524
Total ESAS-r score	0.043	0.005	<0.001	1.044	1.034–1.054
Constant	−2.126	0.095	<0.001	0.119	

*n* = 2071. This model correctly identified 90.9% of those with no nutrition risk and 55.6% of those with malnutrition risk

−2.449 + 0.336 (lack of appetite score) + 0.046 (total ESAS-r score)

When logistic regression models were stratified by gender, results showed that, like the model including all adults, the combination of lack of appetite and total ESAS-r score were the best predictors of malnutrition risk for women (Table 6). For men, the best predictors of malnutrition risk were a combination of lack of appetite, pain, and nausea (Table 7).

**Table 6:** Binary logistic regression model to predict malnutrition risk based on ESAS-r symptom scores among female cancer patients

ESAS score symptoms	B	S.E.	Sig.	Odds ratio	95% CI
Lack of appetite	0.309	0.037	<0.001	1.361	1.266–1.464
Total ESAS-r score	0.048	0.006	< 0.001	1.049	1.036–1.062
Constant	−2.194	0.122	<0.001	0.111	

*n* = 1286. This model correctly identified 90.6% of those with no nutrition risk and 55.6% of those with malnutrition risk

**Table 7:** Binary logistic regression model to predict malnutrition risk based on ESAS-r symptom scores among male cancer patients

ESAS score symptoms	B	S.E.	Sig.	Odds ratio	95% CI
Lack of appetite	0.524	0.050	<0.001	1.688	1.532–1.861
Pain	0.211	0.046	<0.001	1.234	1.135–1.343
Nausea	0.191	0.094	0.042	1.211	1.007–1.457
Constant	−1.977	0.135	<0.001	0.139	

*n* = 785 This model correctly identified 91.8% of those with no nutrition risk and 60.4% of those with malnutrition risk

Tables 8 and 9 show logistic regression models to predict malnutrition risk based on ESAS-r scores for GI cancer patients and non-GI cancer patients. For patients with GI cancers, the best combination of predictors of malnutrition were nausea, lack of appetite, pain, and tiredness. For non-GI patients, the best predictors were lack of appetite and total ESAS-r score.

**Table 8:** Binary logistic regression model to predict malnutrition risk based on ESAS-r symptom scores among GI cancer patients

ESAS score symptoms	B	S.E.	Sig.	Odds ratio	95% CI
Pain	0.177	0.063	0.005	1.194	1.056–1.351
Tiredness	0.108	0.052	0.037	1.114	1.006–1.233
Nausea	0.304	0.126	0.016	1.355	1.058–1.736
Lack of appetite	0.270	0.057	<0.001	1.311	1.172–1.465
Constant	−1.235	0.181	<0.001	0.291	

*n* = 421. This model correctly identified 78.3% of those with no nutrition risk and 70.0% of those with malnutrition risk

**Table 9:** Binary logistic regression model to predict malnutrition risk based on ESAS-r symptom scores among non-GI cancer patients

ESAS score symptoms	B	S.E.	Sig.	Odds ratio	95% CI
Lack of appetite	0.373	0.036	<0.001	1.452	1.353–1.558
Total ESAS-r score	0.047	0.006	<0.001	1.048	1.036–1.060
Constant	−2.452	0.116	<0.001	0.086	

*n* = 1650. This model correctly identified 92.7% of those with no nutrition risk and 54.1% of those with malnutrition risk

The binary logistic regression models showed which combination of ESAS-r parameters best predicted malnutrition, however, using the full regression models to screen for malnutrition in a hospital setting was not deemed to be feasible. Using combinations of ESAS-r parameter cut-offs was deemed to be more feasible. The ROC curves for each individual ESAS-r parameter showed that an appetite score ≥ 1 could be an efficient cut-off to use because, by itself, lack of appetite identified 77.4% of those with malnutrition correctly and only incorrectly flagged 23% of those without malnutrition. Based on the results of the logistic regression models, we examined a combination of the ESAS-r parameters of lack of appetite and total ESAS-r score. We used a lack of appetite score of ≥ 1 and experimented combining that lack of appetite cut-off with total ESAS-r score cut-offs of > 14, >15 or > 16, calculating the sensitivity and specificity for each. These potential total ESAS-r score cut-offs were chosen based on the individual ROC curve for total ESAS-r score. This range of total ESAS-r score values were those deemed most likely to increase sensitivity with minimal compromising of specificity. A combination of an ESAS-r lack of appetite score ≥ 1 and a total ESAS score of > 14, correctly identified 87.9% of the people with malnutrition and incorrectly identified 37.2% of those without malnutrition. Using an ESAS lack of appetite score ≥ 1 and a total ESAS score of > 15, resulted in the % of people with malnutrition identified dropping to 86.9% and the % of those without malnutrition incorrectly identified dropping to 35.2%. Using an ESAS lack of appetite score ≥ 1 and a total ESAS score of > 16, the % of people with malnutrition identified was 84.6% and the % without malnutrition that would be incorrectly flagged was 33.7%.

## Discussion

The results of this study support the association between cancer symptom severity and malnutrition risk in an adult oncology population. ESAS-r symptom scores were found to be significantly higher in patients identified at risk for malnutrition compared to patients not identified at risk for malnutrition. Further, malnutrition risk as measured by the abPG-SGA is significantly and positively correlated to all ESAS-r symptom scores. ROC analyses indicated that the ESAS-r parameters of total ESAS-r score, lack of appetite, tiredness, and wellbeing were most accurate at differentiating between patients identified at risk for malnutrition and patients not identified at risk for malnutrition (AUC = 0.824, 0.812, 0.764, and 0.761 respectively). Based on the ROC analyses, using the individual parameter of lack of appetite score ≥ 1 identified 77.4% of those with malnutrition correctly and only incorrectly flagged 23% of those without malnutrition. When the lack of appetite score is combined with a total ESAS score cut-off of > 14, there is a higher sensitivity of 87.9% although there is a lower specificity with 37.2% of those without malnutrition being incorrectly identified.

Other studies have demonstrated associations between symptoms included on the ESAS-r and malnutrition risk. For example, a 2018 study by Kasvis, Vigano and Vigano (*n* = 512) found that feeling of wellbeing as measured by the ESAS tool was negatively affected by worsening appetite in cancer patients classified as having pre-cachexia, cachexia, and refractory cachexia [53]. Bauer et al. (*n* = 3406) found that hospitalized older adults (aged > 65) that experienced pain were more likely to be at risk of malnutrition than patients without pain [54]. Chabowski et al. (*n* = 257) reported that lung cancer patients classified as malnourished or at risk for malnourishment reported significantly higher levels of pain, anxiety, and depression than patients classified as having a normal nutritional status. There were significant negative correlations between nutritional status and pain (*r* = − 0.65; *P* < 0.001), anxiety (*r* = − 0.68; *P* < 0.001), and depression (*r* = − 0.60; *P* < 0.001) [9]. Additional studies have corroborated the associations between malnutrition risk and depression, pain, and psychological distress [15, 55, 56].

The only research to our knowledge that has aimed to explicitly measure the relationship between ESAS-r scores and malnutrition risk using the abPG-SGA in cancer patients is a preliminary study conducted out of Ottawa, Ontario in conjunction with Ontario Health (Cancer Care Ontario). This study found a statistically significant positive correlation (*r* = 0.502, *p* < 0.0001) between the ‘lack of appetite’ score on the ESAS-r and score on the abPG-SGA [57]. The findings of this study have so far only been disseminated in an internal report, and the results are limited due to small sample size. Further, only one ESAS-r score (lack of appetite) was investigated for its potential relationship with malnutrition risk. Our study builds on the results of this study with a larger sample size and analysis of relationships with all ten ESAS-r parameters including logistic regression models.

As cancer patients in Ontario are asked to complete the ESAS-r at a minimum of once per month when attending visits with their cancer team and up to once per week when having more regular visits, this tool is well-positioned to be used to trigger secondary malnutrition screening throughout the course of treatment. A 2019 study by Diplock et al. based out of Sunnybrook Health Sciences Centre aimed to assess the impact of ESAS implementation for oncology outpatients (Cancer Care Ontario had not yet introduced ESAS-r) by comparing health-related quality of life, patient satisfaction with care, and supportive care service knowledge and utilization between patients screened with ESAS after site implementation and patients who were not screened prior to ESAS implementation. The study found that there was a significant decrease in nausea/vomiting and constipation in ambulatory cancer patients 2 weeks after having completed the ESAS tool. However, there were no significant differences in health-related quality of life, patient satisfaction with care, or knowledge of/access to supportive care between patients who had received ESAS screening and patients who had not. This study concludes that the ESAS tool shows potential to improve symptom management, but standardized pathways to initiate symptom management conversations and facilitate access to appropriate interventions in response to the ESAS should be considered to improve clinical utility [58].

Use of ESAS-r cut-offs to trigger secondary, symptom-specific screening has been seen with the implementation of depression screening after patients score an ESAS-r depression score of 2 at some sites in Ontario. The current research suggests that a similar protocol could be used to identify patients in need of nutrition rescreening based on ESAS-r scores and lack of appetite scores. Cancer centres could use the results of this study to guide nutrition rescreening initiatives.

There are several limitations to this study. First, although both the ESAS-r and abPG-SGA are meant to be administered at first visit to the GRRCC, only 2071 of 3472 patients in the original databases recorded an ESAS-r on the same day as their abPG-SGA. It is not known whether patients who did not complete an ESAS-r from the same day as their abPG-SGA score are different than the patients who did. It is also possible that some patients were missed entirely at first consult and completed neither tool, which could introduce non-response bias. The institutional policy during the time of the data collection was for clinical assists to administer the abPG-SGA to all patients prior to their visit with their care provider at first consult at the GRRCC. This consecutive sampling helps reduce selection bias. However, the data were initially collected with the primary aim of quality improvement, not research. Given that data collection depended on manual delivery of tools, potential reasons that patients may not have completed both tools on the day of intake include human error, staffing variations, workflow disruptions on a given day, and English language proficiency of the patient. Additionally, as this study was conducted retrospectively using existing databases, it is difficult to know whether all patients were truly captured at first consult at the GRRCC. Further, first consult at GRRCC may represent various timepoints within an individual patient’s cancer care journey depending on factors such as the type of cancer, the stage and grade at which the cancer was detected, and the patient’s individualized wishes with respect to their care and treatment team. Patients frequently interact with several services such as medical and radiation oncology at the GRRCC, which could explain why there were 10 patients for which there were two ESAS-r scores completed on the same day. As this study was composed of patients captured at first consult, the relationship between ESAS-r scores and malnutrition risk can only be assessed on a cross-sectional rather than continuous basis. Generalizability of the findings of this study are limited by the population served by the GRRCC which may be different than populations served in other geographic areas. Given that the aim was for patients to be captured at first consult at the GRRCC, the results of this study are most applicable to patients early on in their cancer care, evidenced by the high proportion of patients who did not yet have cancer staging completed at the time of the data collection. While this limits the generalizability of the results, it is also an asset to this study and its practical applications as capturing patients at risk for malnutrition earlier on in their treatment trajectory is favourable to implement proactive rather than reactive nutritional management strategies. The fact that this study preferentially reflects patients early in their treatment journey makes the 33.6% of patients who were found to be at risk for malnutrition especially notable and underscores the importance of implementing malnutrition screening early on in the cancer care treatment journey.

Future directions for this research may include assessing the relationship between ESAS-r symptoms and malnutrition risk on a continuous basis throughout the course of illness as well as incorporating novel symptom screening measures. Future studies could incorporate data captured at several time points over the course of illness and treatment to inform how this relationship may change over time after cancer diagnosis. Further, a revised ESAS measure (ESAS-r+) which asks about three additional symptoms of constipation, diarrhea and sleep has been introduced for the purposes of cancer screening and could be incorporated into future cancer research. When considering the addition of further symptom screening tools, patient preference and the ethical implications of additional screening in cancer patients should always be kept in mind.

## Conclusion

In conclusion, malnutrition risk as measured by the abPG-SGA and symptom severity scores as measured by the ESAS-r are positively and significantly correlated. Utilizing specific cut-offs on the ESAS-r to trigger malnutrition screening could be a viable way to facilitate identification and appropriate management of cancer patients at risk for malnutrition.

## Data Availability

The dataset used and/or analysed during the current study is not publicly available as it is stored on Grand River Hospital’s private server. The dataset is available from the corresponding author on reasonable request.

## Abbreviations

abPG-SGA: Abridged patient-guided subjective global assessment
GRRCC: Grand River Regional Cancer Centre
ESAS-r: Revised Edmonton Symptom Assessment System
PG-SGA SF: Patient-guided subjective global assessment, short form
SGA: Subjective global assessment
ROC: Receiver operating characteristic
AUC: Area under the curve
ESPEN: European society for clinical nutrition and metabolism
